# Mental Health Problems in Girls Who Committed Sexual Offenses: Similarities and Differences Compared to Girls With Non-sex Offenses and Boys With Sex Offenses

**DOI:** 10.3389/fpsyg.2021.721927

**Published:** 2021-12-17

**Authors:** Madleina Manetsch, Rebecca J. Nelson Aguiar, Daniel Hermann, Claudia van der Put, Thomas Grisso, Cyril Boonmann

**Affiliations:** ^1^Department of Forensic Child and Adolescent Psychiatry, Psychiatric University Hospitals (UPK) Basel, Basel, Switzerland; ^2^Department of Psychiatry and Health Behaviors, Augusta University, Augusta, GA, United States; ^3^Research Institute Child Development and Education, University of Amsterdam, Amsterdam, Netherlands; ^4^Center for Mental Health Services Research, Department of Psychiatry, University of Massachusetts Medical School, Worchester, MA, United States; ^5^Child and Adolescent Research Department, Psychiatric University Hospitals (UPK) Basel, Basel, Switzerland

**Keywords:** female juvenile sexual offenders, JSOs, mental health problems, MHP, MAYSI-2

## Abstract

Female juvenile offenders have only recently shifted into the focus of research. Moreover, a specific subgroup, female juveniles who sexually offended (JSO) are greatly overlooked. Therefore, there is a dearth of knowledge regarding the characteristics of female JSOs. The aim of the current study was to explore mental health problems (MHP) of female JSOs in more detail. Moreover, we compared their mental health with female juveniles who committed non-sexual offenses (JNSOs) and male JSOs. The sample comprised 33 female JSOs (Mean age 14.5, SD 1.8), 33 age-matched female JNSOs, and 33 age-matched male JSOs. We used the Massachusetts Youth Screening Instrument-version 2 to examine MHP. Although both internalizing and externalizing MHP were not uncommon in female JSOs, they reported fewer problems than female JNSOs. No differences were found between female and male JSOs. With regard to their mental health profile, female JSOs resemble male JSOs more than female JNSOs. These results should be taken into account in the assessment and treatment of this group. However, more research is needed.

## Introduction

Sexual offenses committed by juvenile females have rarely been the focus of research or clinical practice. This lack of interest is due to various reasons, such as the assumption that females do not commit sexual crimes, the low prevalence of these types of crimes in females, and the belief that sex offenses committed by females are less harmful (Oliver and Holmes, 2015). As Cortoni (2015) noted, offending by females is not a new phenomenon and belittlement of these criminal acts is outdated. Despite the belief that sexual offending behavior is less common in female compared to male juveniles, Slotboom et al. (2011) found that female and male adolescents reported similar rates of sexual aggression against other people. Furthermore, it has been suggested that many of the sexual offenses committed by females remain unnoticed, undetected, or even ignored by law enforcement (Denov, 2004; Hendriks and Bijleveld, 2004; Vandiver and Kercher, 2004). Still, the number of female offenders entering the juvenile justice system, including female juveniles who committed sex offenses (JSOs), has risen alarmingly (Yeater et al., 2015). Especially dire is the fact that the juvenile justice system (i.e., court, criminal justice, and prison), including assessment and treatment, seems to be more tailored toward male offenders than toward female offenders (Plotch et al., 1996). Therefore, more knowledge regarding female juvenile offenders, including female JSOs, is required. Hence, the main aim of the current paper was to examine mental health problems (MHP) in female JSOs. Additionally, we compare MHP in female JSOs to female juvenile non-sexual offenders (JNSOs) and to male JSOs.

Sexual offending behavior in female adolescents is relatively rare. It has been estimated that female adolescents account for about 5 to 10 percent of all juvenile sexual offenses (Matsuda et al., 1989; Lane and Lobanov-Rostovsky, 1997; Righthand and Welch, 2001; Finkelhor et al., 2009; Slotboom et al., 2011). Female JSOs often choose “victims of convenience” (e.g., family members) and their motives are often not of sexual nature (Oliver and Holmes, 2015). Research has shown that female JSOs have histories of abuse and neglect (Vandiver and Teske, 2006), predominantly experienced sexual victimization (Oliver and Holmes, 2015), with some studies reporting prevalence rates exceeding 80% (Schwartz et al., 2006; Mccartan et al., 2011). Other forms of child maltreatment, such as physical abuse, emotional abuse, and/or neglect, were also frequently found (Ray and English, 1995; Kubik et al., 2002; Hickey et al., 2008). Additionally, MHP are highly prevalent in female JSOs (Hunter et al., 1993; Bumby and Bumby, 1997; Mathews et al., 1997; Roe-Sepowitz and Krysik, 2008). For example, in one study (Roe-Sepowitz and Krysik, 2008), almost half of 118 female JSOs had a current mental disorder, received mental health treatment, or took mental health-related medication. Furthermore, 30% reported anger-irritability, 39% depression-anxiety, and 23% suicide ideation problems, and 43% reported traumatic experiences, as measured with the Massachusetts Youth Screening Instrument-2 (MAYSI-2: Grisso and Barnum, 2006). In their summary of the literature, Oliver and Holmes (2015) listed depression, suicidal ideation/attempts, post-traumatic stress disorder, attention-deficit/hyperactivity disorder, and conduct disorder (CD) as frequent mental disorders. In addition, female JSOs often live in dysfunctional families, show inadequate social skills, or have few healthy friendships with their peers (Kubik et al., 2002; Oliver and Holmes, 2015).

The number of studies comparing female JSOs and JNSOs is limited. In one study (Kubik et al., 2002), a sample of 11 girls with sexual offense histories was compared to an age-matched sample of 11 girls with non-sexual victim-involved offense histories. Female JSOs had significantly fewer alcohol and/or drug abuse problems, fighting or aggressive behavior problems, and school problems, than girls in the non-sexual offending group. In an additional study, Kubik and Hecker (2005) compared 11 female JSOs, 12 female JNSOs, and 21 female non-offenders on cognitive distortions about sexual offending. Cognitive distortions, such as “the offender was not responsible for initiating the sexual contact,” were more common in the sexual offending group than in the other two groups. A third study comparing adolescent girls with and without sexually abusive behavior referred to a forensic mental health outpatient setting (Mccartan et al., 2011) found that JSOs were more likely to have a history of abuse, more likely to have a mental disorder, less aggressive, and less self-harmful behavior than JNSOs. Although not statistically significant, mainly due to the large group size difference and the small number of female adolescents with sexually abusive behavior, substance use problems were less common in JSOs. Finally, Van Der Put et al. (2014) compared a sample of 40 girls with sexual offense histories to a sample of 533 girls with non-sexual violent offense histories. They found that female JSOs less often had antisocial friends, family problems (e.g., running away, parental problems, and parenting style), and school problems (e.g., truancy, dropping out, and behavior problems) than female JNSOs. Experienced sexual victimization outside the family and social isolation, however, were more common in the sexual offending group. These results generally suggest that female JSOs show less externalizing, but more internalizing MHP compared to female JNSOs.

Research comparing female and male JSOs is also scarce. In general, female and male JSOs were often found to be similar regarding their criminal histories and psychosocial problems (Bumby and Bumby, 1997; Mathews et al., 1997; Kubik et al., 2002; Van Der Put et al., 2014). Mathews et al. (1997), for example, reported similar rates of prior mental health treatment, runaway behavior, and attempted suicide. Similarly, Van Der Put et al. (2014) found no differences on criminal history, MHP, family problems, peer problems, and school problems. Despite this high degree of similarity, both groups seem to differ in prevalence and context of adverse childhood experiences (Vandiver, 2010). Female JSOs often showed higher rates of experienced physical (Mathews et al., 1997; Kubik et al., 2002) and sexual abuse (Mathews et al., 1997; Kubik et al., 2002; Schwartz et al., 2006) compared to male JSOs. They were also more likely to be sexually victimized by multiple perpetrators and experienced more severe and longer lasting abuse then their male counterparts (Mathews et al., 1997; Schwartz et al., 2006). With regard to their own offending behavior, female JSOs were found to be younger at the time of the offense, had younger victims, were more likely to co-offend, and more likely be involved in incidents with multiple victims (Finkelhor et al., 2009). Regarding MHP, higher rates of alcohol and/or drug abuse were found in female JSOs compared to male JSOs (Mathews et al., 1997; Van Der Put et al., 2014).

The treatment of JSOs has been the focus of a large part of research for quite some time (Worling, 1998; Seto and Lalumiere, 2010; Dwyer and Letourneau, 2011). Involved professions and researchers agree on the fact that a “one size fits all” approach falls short in view of the heterogeneity of this offender group. For female JSOs, it is important to understand to what extent they differ from both female JNSOs and male JSOs (Ganon et al., 2010), as this could guide treatment. To the best of our knowledge, there are only two studies in which female JSOs were compared to both female JNSOs and male JSOs with regard to MHP (Kubik et al., 2002; Van Der Put et al., 2014). However, the sample of the study of Kubik et al. (2002) was small, which limits the generalizability. Results of the study of Van Der Put et al. (2014) did not examined MHP in detail.

The aim of the present study was to gain better insight into specific MHP of female JSOs by comparing them with both female JNSOs and male JSOs. More insight into similarities and differences in MHP between these groups could help us to improve the treatment of these juvenile female offenders.

## Materials and Methods

### Procedures

We selected our sample from a study sample originally collected by Maney (2011). Maney’s sample included scores made by juvenile offenders on the MAYSI-2 (Grisso and Barnum, 2006). At the time of data collection for Maney’s study, 451 juvenile justice facilities in the United States were administering the MAYSI-2 to all youths at entry to their facilities, using a software called MAYSIWARE (Maney and Grisso, 2006). When Maney contacted these facilities, 65 sites provided their MAYSIWARE databases for research use, and these databases were merged to create a single nationwide MAYSIWARE database with 54,716 MAYSI-2 administrations. These sites spanned 17 U.S. states and represented juvenile intakes in probation, detention, and correctional facilities. The MAYSIWARE software program records youth demographics (age, gender, race, and ethnicity), offense information (up to six current charges/offenses leading to involvement in the juvenile justice system), type of facility (probation, detention, or corrections), and adjudication status of the youth (pre-trial or post-adjudication).

### Participant Selection

**Figure 1:**
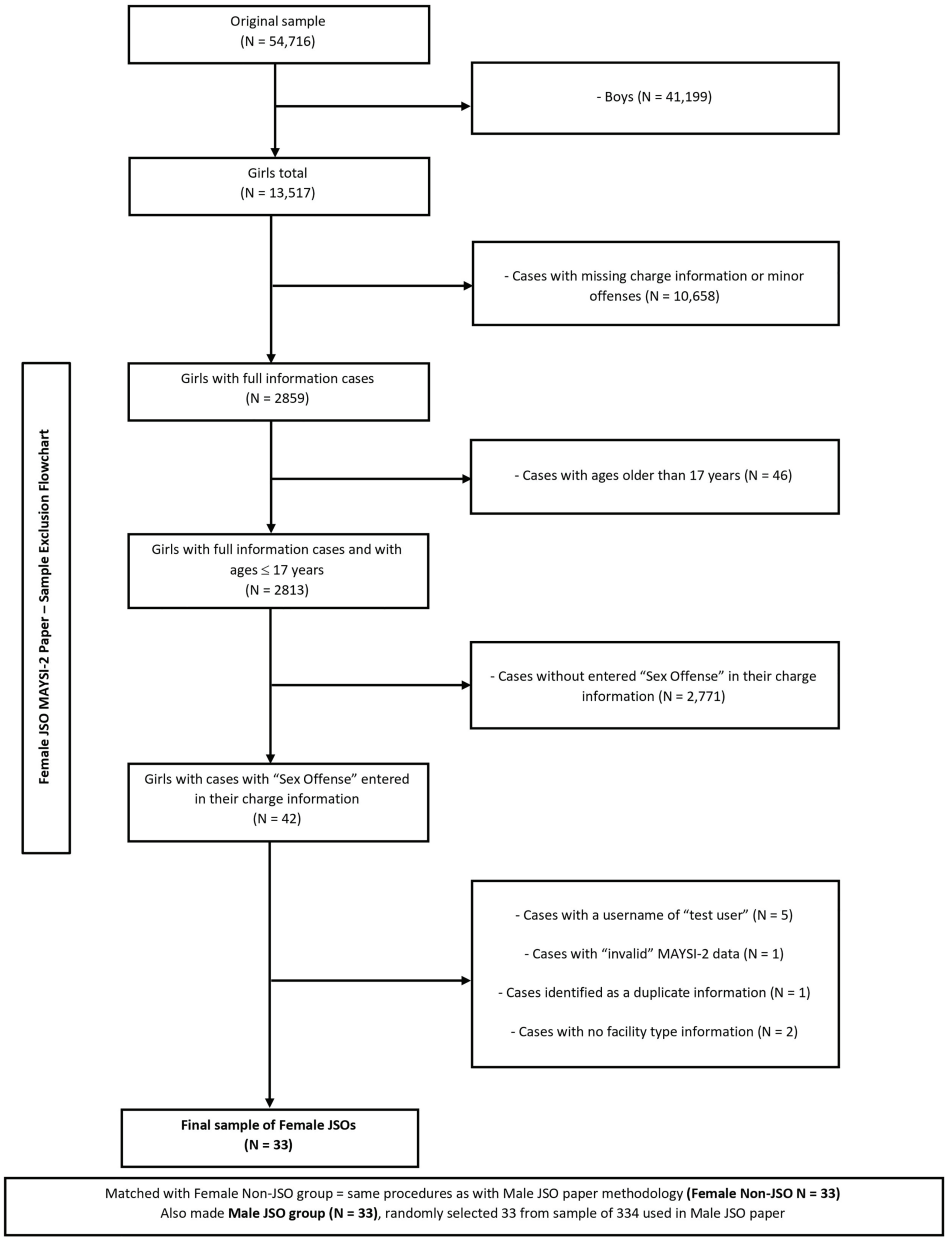
Flowchart illustrating how the final sample was selected and the number of cases resulting after each step of applying the exclusion criteria.

A number of exclusion criteria were used to create our sample from Maney’s MAYSIWARE database (see Figure 1). First, the sample of female JSOs needed to be identified. There were 13, 517 cases of female adolescents from the original sample. In order to categorize our final sample into sexual offenders or non-sexual offenders, cases with missing charge or offense information were excluded, as were cases with minor charges or convictions (e.g., status offenses, breaches of orders; *n* = 10, 658), resulting in a sample of *n* = 2, 859. While the MAYSI-2 was validated for 12- to17-year-olds, only cases with ages above 17 years were excluded from the sample (*n* = 46), in order to retain as many cases of female JSOs as possible, without keeping cases closer to adulthood (i.e., 18 and older) than adolescence. This resulted in a sample of girls with full information cases and ages ≤17 years of *n* = 2, 813.

Youth were considered to be JSOs when their MAYSI-2 offense information contained at least one sex offense (e.g., sexual abuse of children, rape, or sexual assault) and were considered to be JNSOs when none of their MAYSI-2 offense information showed a sex-related offense. With a working database of 2, 813 female adolescent cases, 42 youth cases had “sex offense” entered under charge information and were therefore classified as female JSOs. A total of nine cases were excluded due to user information entered as “test case” (*n* = 5), missing information on facility type (*n* = 2), invalid MAYSI-2 data (*n* = 1), and a final case was excluded after being identified as a duplicate administration. Therefore, the final female JSO sample consisted of 33 cases.

The 2, 813 female cleaned cases were used to select a matched sample of 33 general female offenders. The male JSO matching group (*n* = 33) was randomly selected from a sample of 334 male JSOs from the original database, created, and utilized in a previous paper comparing male JSOs and JNSOs (Boonmann et al., 2016b).

### Participant Characteristics

The sample consisted of 33 female JSOs, 33 female JNSOs, and 33 male JSOs. Participants across groups were matched on age, race/ethnicity, and type of facility. The ages in the sample ranged from 11 to 17 years old, with an average age of 14.5 years (*SD* = 1.8; see Table 1). The majority of youth were White Non-Hispanic (52.5%), 25.3% were African-American, and 22.2% were Hispanic. Most juvenile offenders were in detention or correctional facilities (69.7%) compared to intake probation (30.3%) at the time of data collection. Thus, this must be taken into account regarding the different settings. Even though participants were not matched on disposition, the three groups did not significantly differ on adjudication status (i.e., pre-trial or post-adjudication), *χ*2 (2, *n* = 99) = 2.85, *p* = 0.24.

**Table 1:** General Descriptives.

*N*	Total	Female JSO	Female NJSO	Male JSO	*F*/*χ*^2^	*P*
	99	33	33	33		
Age						
Mean (*SD*)	14.5 (1.8)	14.4 (1.8)		14.6 (1.7)	*F* = 0.21	0.813
Range	11–17			12–17		
Gender						
Male	33.3%					
Female	66.7%	100%		100%		
Race						
Black	25.3%	18.2%		39.4%	*χ*^2^ = 5.68	0.225
White	54.6%	63.6%		39.4%		
Other	19.2%	18.2%		15.2%		
Ethnicity						
Non-Hispanic	77.8%	78.8%		81.8%	*χ*^2^ = 0.82	0.664
Hispanic	22.2%	21.2%		18.2%		
Facility Type						
Probation	30.3%	36.4%		12.7%	*χ*^2^ = 16.2	0.003
Detention	64.6%	63.6%		72.7%		
Corrections	5.1%	–		15.2%		

### Measures

The MAYSI-2 (Grisso and Barnum, 2006) is a 52-item self-report mental health screening tool created for use in the juvenile justice system for youth aged 12- to17-years-old. Youth report whether each item, referring to thoughts or feelings, has been true for them in the past few months. The MAYSI-2 consists of seven scales (alcohol/drug use, angry-irritable, depressed-anxious, somatic complaints, suicide ideation, thought disturbance, and traumatic experiences). The thought disturbance scale has been developed for boys only. Raw scores are calculated and used to identify young people whose self-report of particular psychological and emotional symptoms reaches the caution or warning level, which have been based on national norms and past research. A score above the caution cutoff suggests that the reported problem falls in the clinically significant range, while the warning cutoff reflects scores higher than those rendered for 90% of youth in the normative sample (Grisso and Barnum, 2006). They can be used by clinicians to determine which young people need further assessment. As a screening tool, it is not intended to be diagnostic of mental disorders. All scales have cutoff scores with the exception of the traumatic experiences scale, which is not considered a clinical scale, but rather a reported history of experienced trauma. The MAYSI-2 has been well researched, and studies have found it to have adequate reliability and validity (Grisso et al., 2012).

Although it differs by facility type (e.g., probation or corrections) and by facility resources (private solo administration or group format), most youth complete the MAYSI-2 within the first 24–72 h of admission to a facility. Facilities may also re-administer the tool if a youth leaves the facility and comes back (e.g., a court date) or if a youth displays behavior causing concern (e.g., self-harm).

### Data Analysis

First, descriptive statistics of the MAYSI-2 were performed, which included computation of the mean MAYSI-2 scale scores and the percentage of youth above the caution and warning cutoffs. Next, differences in mental health scores between the female JSOs, female JNSOs, and male JSOs were tested by means of ANOVA. The level of statistical significance for all statistical tests was set at *p* ≤ 0.05. Subsequently, effect sizes were calculated using Cohen’s d. The classification provided by Cohen (1988) was used to interpret the magnitude of the effect sizes (small: *d* = 0.20–0.49; medium: *d* = 0.50–0.79; large *d* = 0.80+).

## Results

In total, 51.5% of female JSOs reported somatic complaints above the caution cutoff. Furthermore, 39.4% reported depressed-anxious problems and 36.3% angry-irritable problems in the clinically significant range (score above the caution cutoff). Finally, 24.3% reported above the caution cutoff on suicide ideation and 12.1% on alcohol and drug use problems. As mentioned before, there is no thought disturbance subscale for females and the traumatic experience subscale has no caution or warning cutoff points (Table 2).

**Table 2:** MAYSI Descriptives for female JSO.

	Min.	Max.	Caution^*^% (*N*)	Warning% (*N*)
Alcohol/Drug Use	0	6	9.1 (3)	3.0 (1)
Angry-Irritable	0	8	33.3 (11)	3.0 (1)
Depressed-Anxious	0	6	33.3 (11)	6.1 (2)
Somatic Complaints	0	6	42.4 (14)	9.1 (3)
Suicide Ideation	0	5	15.2 (5)	9.1 (3)
Thought Disturbance	–				–	–	–
Traumatic Experience	0	5	–		–

* “Caution” means over the caution cutoff but below the warning cutoff.

Female JSOs scores were significantly lower than those of female JNSOs on angry-irritable problems, depressed-anxious problems, and somatic complaints (see Table 3). Furthermore, female JSOs did not significantly differ from male JSOs on any MAYSI-2 scale (see Table 3).

**Table 3:** Comparison female JSOs, female NJSOs, and male JSOs.

	Female JSO	Female JNSO				Female JSO	Male JSO			
	*M*(*SD*)	*M*(*SD*)	*t*	*P*	*d*	*M*(*SD*)	*M*(*SD*)	*t*	*P*	*d*
Alcohol/Drug Use	0.9 (1.7)	1.9 (2.7)	1.70	0.093	0.044	0.9 (1.7)	1.0 (2.1)	0.13	0.898	0.052
Angry-Irritable	3.2 (2.2)	4.9 (2.9)	2.78	0.007	0.066	3.2 (2.2)	2.5 (2.7)	−1.14	0.259	0.284
Depressed-Anxious	2.0 (1.7)	3.2 (2.7)	2.09	0.041	0.053	2.0 (1.7)	1.7 (1.9)	−0.60	0.551	0.133
Somatic Complaints	2.3 (1.8)	3.4 (2.0)	2.28	0.026	0.058	2.3 (1.8)	2.4 (1.7)	0.29	0.775	0.057
Suicide Ideation	0.6 (1.3)	1.2 (1.9)	1.71	0.092	0.037	0.6 (1.3)	0.6 (1.3)	0.000	1.00	0.000
Traumatic Experience	1.4 (1.5)	2.1 (1.8)	1.73	0.088	0.042	1.4 (1.5)	1.6 (1.5)	0.66	0.513	0.133

## Discussion

The main aim of the current paper was to gain better insight into MHP of female JSOs and to compare them with both female JNSOs and male JSOs. Our results showed that both internalizing and externalizing MHP were not uncommon in female JSOs, however, to a lesser extent than their female counterparts without sexual offenses (JNSOs). Compared to male JSOs, no differences were found.

Our results, which showed that JSOs have fewer MHP than JNSOs, are largely consistent with previous research (Kubik et al., 2002; Mccartan et al., 2011; Van Der Put et al., 2014; Van Der Put, 2015). Female JSOs were found to have less externalizing MHP than female JNSOs (Kubik et al., 2002; Mccartan et al., 2011; Van Der Put et al., 2014; Van Der Put, 2015). Results regarding internalizing MHP are somewhat less clear. In line with our results (i.e., less internalizing MHP in female JSOs than in female JNSOs), Mccartan et al. (2011) also found less self-harm. Van Der Put et al. (2014), otherwise, found more social isolation in females JSOs than in female JNSOs. Internalizing MHP are a very broad concept. In the MAYSI-2, anxiety and depression are summarized under one category. Given the increasing interest in internalizing MHP among juvenile offenders and taking into account gender differences as well as differences between offenders with and without sex offenses in this domain, more in-depth research on underlying internalizing MHP among female JSOs in general, as well as relative to JNSOs, is preferred. In contrast to our results, (i.e., no significant differences in traumatic experiences between both groups) other studies found more own sexual victimization in female JSOs (Mccartan et al., 2011; Van Der Put et al., 2014). A possible explanation could be that, in the current study, traumatic experiences were examined combined, whereas other studies specifically examined sexual abuse (Mccartan et al., 2011; Van Der Put et al., 2014). More research regarding traumatic experiences in female JSOs, potentially in combination with internalizing MHP, is warranted.

Our results regarding MHP in female JSOs compared to male JSOs are also generally in line with previous research (Bumby and Bumby, 1997; Mathews et al., 1997; Kubik et al., 2002; Van Der Put et al., 2014), which showed that female and male JSOs are more similar than different. However, unlike these studies which showed that female JSOs were overall more likely to suffer from sexual abuse, physical abuse, emotional abuse, and neglect than male JSOs, we found no significant differences in traumatic experiences between the two groups. The same argument as mentioned above could apply; in our study, we were not able to subdivide traumatic experiences into specific types of traumatic experiences, such as sexual abuse, physical abuse, emotional abuse, and neglect (Ray and English, 1995; Mathews et al., 1997; Kubik et al., 2002). More in-depth research regarding abuse and neglect and MHP are of great importance, as the relations between childhood sexual abuse and sexual antisocial behavior might be influenced by MHP more than is apparent at first sight (Boonmann et al., 2016a).

Based on our results, a mixed-gender approach for clinical practice could be reflected on. This mixed-gender approach, instead of a focus on “female” in females JSOs, is also in line with the results of Van Der Put (2015) who found more protective and fewer risk factors regarding school, relationships, and family in the female JSO compared to the female JNSO group. This underlines the fact that female JSOs and female JNSOs seem to have different treatment needs. Given the lack of validated treatment programs for female JSOs, it could be argued that (parts of) male JSOs treatment approaches could be applied for the treatment of female JSOs, although adjusted to suit a female perspective (e.g., by including examples of female JSOs in treatment manuals). In addition, trauma-informed care, which is also receiving increasing attention within the forensic field (Branson et al., 2017; Schmid et al., 2020), could not only benefit female, but also male offenders. Nevertheless, there is a risk for a “one size fits all” approach, and despite the many similarities, the diversity of the group, however, should not be overlooked. Therefore, further research examining the existing variations more in-depth in order to better understand female JSOs is warranted.

The results of the current study should be interpreted in the light of some limitations. First, the current study consisted of a small sample and it is possible that statistically significant results went undetected due to limited statistical power. Although this is an important limitation, it unfortunately is often a reality in this field of research. However, given the lack of research on female JSOs the findings of this study significantly contribute to the literature. More research with larger samples is critical to confirm the results of this study and expand the body of research. In addition, JSOs are a very heterogeneous group (Van Wijk and Boonmann, 2017). This also seems to be the case for female JSOs (Van Der Put, 2013; Van Der Put et al., 2013). However, due to the limited sample size, we were not able to examine subgroups or female JSOs. More research in subgroups, for example based on offender age, victim age, and type of offense (hands-on vs. hands-off, number of offenders), will help us better understand this group of JSOs. Second, we classified female JSOs based on their MAYSI-2 offense history and not on official records. Hence, some young people could be included in (sex offense in MAYSIWARE, but acquitted of this offense) or excluded from (history of sexual offending, unknown to MAYSIWARE) this group incorrectly. Third, the MAYSI-2 is a self-report instrument and information reported by adolescents could have been biased due to factors such as social desirability, feelings of shame/embarrassment stemming from the stigmatization of having mental health concerns, and cultural norms or other reasons to avoid expectations surrounding discussing and reporting mental health symptoms. In addition, it should be taken into account that the MAYSI-2 is a screening tool and not a diagnostic classification. Our results therefore only refer to MHP and not to mental disorders.

## Conclusion

Our results demonstrated that although both externalizing and internalizing MHP were not uncommon in female JSOs, they reported fewer problems than female JNSOs. No differences were found between female and male JSOs. With regard to their mental health profile, female JSOs resemble male JSOs more than female JNSOs. This result could guide clinical conduct and treatment interventions.

## Data Availability Statement

The original contributions presented in the study are included in the article/supplementary material, and further inquiries can be directed to the corresponding author.

## Ethics Statement

The sample was constructed from archival intake records consisting of data sets that were obtained from 283 juvenile justice facilities in 19 U.S. states during a three-year period. The MAYSI-2 had been administered to these youths at the time of their intake into the facilities as part of normal, clinical procedures and use, not for research purposes, when they entered juvenile justice facilities. Therefore, informed consent at the time the MAYSI-2 was administered was not required. Later, when facilities were invited to volunteer their archival data sets for research use, the identity of all youths was removed from each case before the data sets were transferred to the researchers. Compilation of the data base for research use under these conditions (without individual informed consent by youths) was reviewed and approved by the Internal Review Board of the University of Massachusetts Medical School as complying with U.S. federal regulations for ethical protection in research involving human subjects.

## Author Contributions

MM and CB wrote the first draft of this manuscript. The data set was collected by TG and colleagues, and the data analyses were conducted by RN and CB. All authors participated in the writing of the manuscript and were involved in designing and planning of the study. All authors contributed to the article and approved the submitted version.

## Conflict of Interest

The authors declare that the research was conducted in the absence of any commercial or financial relationships that could be construed as a potential conflict of interest.

## Publisher’s Note

All claims expressed in this article are solely those of the authors and do not necessarily represent those of their affiliated organizations, or those of the publisher, the editors and the reviewers. Any product that may be evaluated in this article, or claim that may be made by its manufacturer, is not guaranteed or endorsed by the publisher.
